# Why Do Men Report More Opposite-Sex Sexual Partners Than Women? Analysis of the Gender Discrepancy in a British National Probability Survey

**DOI:** 10.1080/00224499.2018.1481193

**Published:** 2018-07-25

**Authors:** Kirstin R. Mitchell, Catherine H. Mercer, Philip Prah, Soazig Clifton, Clare Tanton, Kaye Wellings, Andrew Copas

**Affiliations:** 1 MRC/CSO Social and Public Health Sciences Unit, Institute of Health and Wellbeing, University of Glasgow; 2 Centre for Sexual Health, Public Health, and Policy, London School of Hygiene and Tropical Medicine; 3 Department of Infection and Population Health, University College London

## Abstract

In a closed population and defined time period, the mean number of opposite-sex partners reported by men and women should be equal. However, in all surveys, men report more partners. This inconsistency is pivotal to debate about the reliability of self-reported sexual behavior. We used data from the third National Survey of Sexual Attitudes and Lifestyles (Natsal-3), a probability sample survey of the British population, to investigate the extent to which survey sampling, accounting strategies (e.g., estimating versus counting), and (mis)reporting due to social norms might explain the inconsistency. Men reported a mean of 14.14 lifetime partners; women reported 7.12. The gender gap of 7.02 reduced to 5.47 after capping the lifetime partner number at the 99th percentile. In addition, adjusting for counting versus estimation reduced the gender gap to 3.24, and further adjusting for sexual attitudes narrowed it to 2.63. Together, these may account for almost two-thirds of the gender disparity. Sampling explanations (e.g., non-U.K.-resident partners included in counts; sex workers underrepresented) had modest effects. The findings underscore the need for survey methods that facilitate candid reporting and suggest that approaches to encourage counting rather than estimating may be helpful. This study is novel in interrogating a range of potential explanations within the same nationally representative data set.

Accurate reporting of sexual partners is crucial to a wide range of sexuality research, including measuring trends in sexual behavior assessing individual risk of sexually transmitted infections (STIs) and estimating the rate and modeling the impact of STI/human immunodeficiency virus (HIV) transmission in a population.

In a relatively closed population, the mean number of opposite-sex partners per unit of time reported by men should be similar to that of women, particularly over short time periods (Wadsworth, Johnson, Wellings, & Field, 1996). Although the gap has narrowed over recent decades, surveys across the world find that men typically report about twice as many lifetime partners as women (Mercer et al., 2013; Todd et al., 2009). This inconsistency has long vexed researchers and has underpinned concerns about the veracity of self-reported sexual behavior in general. The discrepancy also provides a key exemplar to investigate (albeit indirectly) validity and bias in surveys of sexual behavior.

There are three approaches to explaining the gap. The first focuses on sampling explanations, such as underrepresentation of sex workers, age mixing, and inclusion of partners who are nonresident in the population (Wadsworth et al., 1996). Such explanations derive from statistical adjustment to investigate hypothesized sampling bias. Brewer and colleagues (2000), for example, suggested that the gender discrepancy could be eliminated by adjusting for underrepresentation of sex workers in population surveys.

The second explanation focuses on gender differences in accounting strategies and recall, observing that less accurate estimation strategies are associated with higher numbers (Bogart et al., 2007). A female tendency to enumerate (count instances) leads to lower estimates, while a male tendency to approximate (Brown & Sinclair, 1999) and to report large round numbers (Wiederman, 1997) leads to overestimates. A small literature has also focused on what is counted and on gendered differences in how sexual partners are defined (Jeannin, Konings, Dubois-Arber, Landert, & Van Melle, 1998; Sanders & Reinisch, 1999), suggesting a higher propensity among men to include nonpenetrative sex partners in their total count.

The third explanation focuses on misreporting due to intentional or unintentional “false accommodation” to perceived gendered norms and expectations (Fisher, 2013; Jonason & Fisher, 2009). Fear of social disapproval for transgressing gender norms may lead men to overreport and women to underreport their lifetime partners (Alexander & Fisher, 2003). Experimental manipulation of survey conditions suggests the gender discrepancy is narrower when participants perceive greater privacy (e.g., in self-complete versus interviewer-administered surveys; Tourangeau & Smith, 1996) and also when they think that lying might be detected (Alexander & Fisher, 2003). Overheard (staged) conversations expressing conservative or permissive norms (Fisher, 2009), as well as the gender of the researcher (Fisher, 2007), have also been shown to affect reporting, particularly among men and women who adhere more strongly to gender stereotypes.

All three explanatory approaches are credible and have some empirical support, so it seems reasonable to conclude that they could all play a potential role in accounting for the gender discrepancy. Currently, we do not know which explanation is primary, because the empirical data typically derive from small and/or unrepresentative studies that cannot be directly compared. Understanding the relative contribution of these competing explanations requires concurrent comparison within the same data set. Brown, Schweickart, Sinclair, and Moore (2017) recently considered accounting and social desirability explanations within the same large data sets, but to our knowledge, our study is the first attempt to compare all three types of explanation simultaneously. We used data from the third National Survey of Sexual Attitudes and Lifestyles (Natsal-3) to investigate all three types of explanation: (a) sampling, (b) accounting strategies, and (c) conformity to gendered norms. We focused on lifetime as the period in which the largest gender discrepancy emerges and in which issues of recall bias and accounting strategies are most pertinent.

## Method

### Participants and Procedure

We present data from Natsal-3, a stratified probability sample survey of 15,162 men and women aged 16 to 74 years in Britain, interviewed between September 2010 and August 2012 (http://www.natsal.ac.uk). We used a multistage, clustered, and stratified probability sample design. We weighted the data to adjust for unequal selection probabilities and poststratified the sample to match the 2011 census by age, sex, and region (Mercer et al., 2013).

Participants were interviewed at home, by a trained interviewer, using computer-assisted face-to-face interviews and computer-assisted self-interviews (CASIs) for the more sensitive questions (Mercer et al., 2013).

The response rate was 57.7% (of all addresses known or estimated to be eligible). Details of the survey methodology are published elsewhere (http://www.natsal.ac.uk; Mercer et al., 2013). Natsal-3 was approved by Oxfordshire Research Ethics Committee A. Participants provided oral informed consent for interviews.

### Measures

Survey measures and time frames are shown in Box 1.

**Box 1.** Self-Report and Face-to-Face Measures From Natsal-3 Used in Analysis

**CASI (Self-Report) Measures**	**Response Options (and Recoding)**
*CASI: Lifetime time frame*	
“Altogether, in your life so far, how many (*women/men*) have you had sexual intercourse with (vaginal, oral, or anal)?” *Note*: Question asked of all women and men regardless of sexual orientation. Analysis includes individuals reporting 0 partners.	Number keyed in. *Note*: Values capped at the 99th percentile (110 for men, 50 for women) in the analysis, i.e., those in excess replaced by the 99th percentile.
Participants reporting five or more lifetime partners: “Which of these best describes how you worked out that answer?”	Response options: 1. I just knew the number. 2. I remembered each partner and counted them up. 3. I estimated or guessed the number. 4. I remembered some partners and then added on an estimated number for others. *Options 1 and 2 are categorized as counting strategies. Options 3 and 4 are categorized as estimating strategies*. *Note*: Analysis showed that, among both men and women, participants who said that they had “remembered some” reported a similar mean number of partners as those who said that they had “estimated or guessed.” In analysis, participants reporting less than five lifetime partners were assumed to have counted, not estimated.
“In your lifetime, how many different *(men/women [opposite sex])* have you paid money for sex?”	Number keyed in.
*CASI: Last five years time frame*	
“Altogether, in the last five years, how many (*women/men*) have you had sexual intercourse with?”	Number keyed in.
“In the last 5 years, have you had sex for the first time, here in the U.K., with anyone who normally lives outside the U.K.?” *Include anyone who was visiting the U.K. or living here for a while*. If yes: “In the last five years, how many people who normally live outside the U.K. did you have sex with for the first time in the U.K.?”	Yes/no. Number keyed in.
“In the last five years, have you had sex with anyone for the first time while you were in any country outside the U.K.?” If yes: “In the last five years, how many people did you have sex with *for the first time* while you were in any country outside the UK?”	Yes/no. Number keyed in. *Note on analysis*: New partners abroad who were U.K. residents were excluded (37%). Among participants reporting new sexual partners from multiple countries while abroad, it was not possible to identify which were U.K. residents, and all these partners were excluded.
*CASI: Last year time frame*	
“Altogether, in the last year, how many (*women/men*) have you had sexual intercourse with?”	Number keyed in.
“Were there any (women/men) you had only oral sex with and never vaginal (or anal) sex?” If yes: “How many different (*women/men*) in the last year did you have *only oral sex* with and *never* vaginal (*or anal*) sex?” “Previously, you said you had sex with (*previous answer inserted*) (*woman/women/man/men*) in the last year. Does this number include the (*woman/women/man/men*) you *had only oral sex with*?”	Yes/no. Number keyed in. Response options: 1. Yes, (*all/both*) included. 2. No, did not include (*all of them/both of them*).
**Face-to-face measures**	
“Tell me what your views are about the following sexual relationships:” “A married person having sexual relations with someone other than his or her partner?” “And what is your opinion about a person having one-night stands?”	Response options on a card provided to participants: 1. Always wrong 2. Mostly wrong 3. Sometimes wrong 4. Rarely wrong 5. Not wrong at all “Depends/Don’t know” not given on card but recorded by interviewer if participant gives this response. *Note*: So as not to exclude participants, individuals reporting *Depends/Don’t know* (269 for the first attitude and 641 for the second) were recoded to the middle option on the Likert scale (*Sometimes wrong*).

### Analysis

We included all participants (men [unweighted, weighted]: 6,023, 7,170; women [unweighted, weighted]: 8,530, 7,323) except those with missing number of partners (< 5%). Participants were included regardless of sexual orientation, because individuals identifying as gay or lesbian may also report at least one opposite-sex partner. As long as men and women with no opposite-sex partners are included in the count, the total number of partnerships (and therefore the mean number of lifetime partners) should be equal, regardless of how people identify.

Using data from Natsal-3 (including previously published data), and grouping by explanatory approach, we present the logic for each inclusion/exclusion or adjustment to the data. We present estimates of the difference in mean number of partners between men and women after applying each exclusion to demonstrate its impact, and use linear regression including adjustment factors as covariates to demonstrate the impact of adjustments. The use of three different time periods (past year, past five years, lifetime) reflects the reporting periods used in the survey (Box 1).

## Results

The demographic characteristics of the sample are summarized elsewhere (Mercer et al., 2013).

### Sampling Explanations

#### Non-U.K.-resident sexual partners

Sexual partners who usually live abroad were ineligible for Natsal. In Natsal-3 (Tanton et al., 2016), men were more likely than women to report having had *new* partners while abroad in the five years prior to interview (8.3% of men versus 4.5% of women).

#### Paid-for partners

In Natsal-3, 10.8% of men reported ever having paid for opposite-sex sex, compared with 0.1% of women (*M* [*SD*], number of paid partners reported by men was 5.94 [12.66]; among women [*n* = 10], 11.41 [30.14]). Among these participants, 35.2% of men and 63.1% of (the 10) women reported that they had excluded these paid partners from their total number of lifetime partners. It has been suggested that full-time sex workers are underrepresented in national surveys (Brewer et al., 2000). Around 26 sex workers would be expected in the sample of 8,530 women, but Natsal-3 did not ask women whether they had been paid for sex.^1^1Based on estimate of 65,000 sex workers in U.K. by the U.K. Network of Sex Work Projects (UKNSWP) mapping exercise cited in Toynbee Hall (2009) Statistics and Prostitution in London and the United Kingdom and 2010 population estimate of 22,352,376 women aged 16–74 (Office for National Statistics; https://www.ons.gov.uk/).

### Accounting Explanations

#### Capping extreme values

Previous research suggests that men report extreme numbers of lifetime partners more often than women do (Jeannin et al., 1998). In Natsal-3, the 99th percentile was 110 partners for men but only 50 for women.

#### Accounting strategy

**Figure 1.**
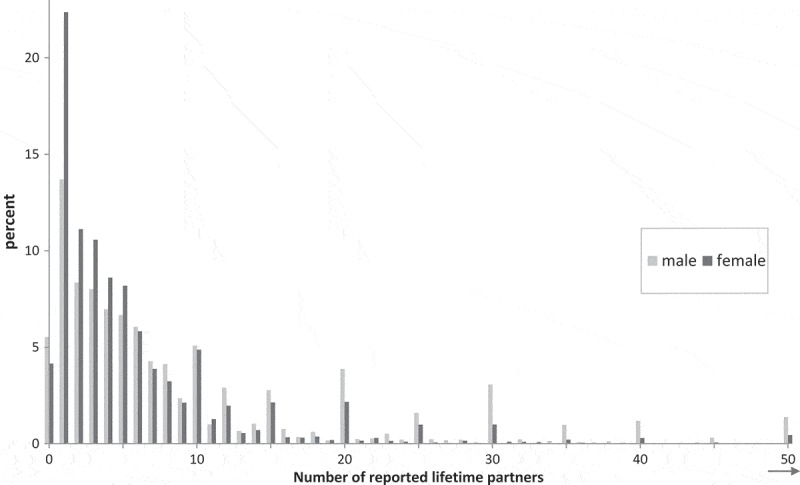
Reported number of opposite-sex partners in lifetime (Natsal-3) by gender (truncated at 50 partners). Denominator is all aged 16 to 74 with data for number of opposite-sex partners in lifetime (*n* = 6,028 men, *n* = 8,530 women); 265 men and 339 women excluded due to missing data.

In Natsal-3, a higher proportion of men than women reported that they had estimated their lifetime number; among those reporting five to nine partners, 24.1% of men estimated, compared with 15.0% of women; while among those reporting 10 or more partners, 63.1% of men said they estimated, compared with 52.1% of women. Consistent with previous research (Tourangeau & Smith, 1996), the use of estimation strategies is evident in a tendency to report partner numbers ending in 0 and 5, particularly with higher numbers of partners (Figure 1). Furthermore, accounting strategy was associated with the number of partners reported. Men who said they had counted reported a mean of 6.7 partners (6.3 to 7.1), while those who estimated reported a mean of 33.7 (26.7 to 40.6). Equivalent means for women were 4.5 (4.4 to 4.7) and 21.9 (16.9 to 26.9).

#### Gendered difference in what counts as a partner

In Natsal-3, men were more likely than women to exclude oral-sex-only partners in their total number of partners (33.1% of men versus 23.4% of women). At the same time, a significantly larger proportion of men than women reported having had oral-sex-only partners in the past year (10.2% of men versus 6.8% of women).

### Conformity to Gendered Norms

#### Sexual attitudes

Natsal-3 has previously reported that women express significantly more conservative attitudes toward casual sex and nonexclusive sex (Mercer et al., 2013). Women were *less* likely to view one-night stands as *Not wrong at all* (9.3% versus 17.5%) and they were *more* likely to view a “married person having sexual relations with someone other than his or her partner” as *Always wrong* (65.0% versus 57.2%) (Mercer et al., 2013). Both attitudes were significantly associated with reporting of sexual partners (*p* < 0.00001). For example, 36.6% of men and 23.8% of women reporting five or more partners in the past year considered casual sex as *Not wrong at all*, compared with 15.0% of men and 8.8% of women who reported just one partner during this time.

### Impact of Inclusions/Exclusions and Adjustments on the Difference in Mean Numbers of Partners Reported by Men and Women

Table 1 shows the gender difference in mean number of reported partners over three time periods and the impact of including/excluding participants based on factors hypothesized to affect the reported means.

**Table 1.** The Impact of Including/Excluding Different Types of Partners, Capping, and Adjusting for Attitudes on the Total Number of Opposite-Sex Partners Reported by Men and Women: Last Year, Past Five Years, and Lifetime

	Men	Women	Difference Between Means	95% Confidence Interval
	*M* (*SD*)	*M* (*SD*)		
**Last year**				
Overall	1.27 (1.99)	1.04 (2.83)	0.23	(0.14 to 0.32)
After including oral-only partners	1.36 (2.20)	1.07 (2.93)	0.28	(0.18 to 0.38)
Unweighted, weighted denominator	6,048, 7,181	8,540, 7,330		
**Last five years**				
Overall	2.66 (5.64)	1.84 (8.12)	0.82	(0.55 to 1.09)
After excluding new partners from outside of the U.K. but while in U.K.	2.60 (5.53)	1.81 (7.82)	0.78	(0.53 to 1.04)
After excluding new partners while outside of the U.K.	2.45 (5.36)	1.76 (7.87)	0.69	(0.43 to 0.95)
After excluding both	2.43 (5.31)	1.76 (7.58)	0.67	(0.43 to 0.92)
Unweighted, weighted denominator	6,067, 7206	8,560, 7353		
**Lifetime**				
Overall	14.14 (63.66)	7.12 (34.62)	7.02	(4.97 to 9.08)
After 99th percentile capping	11.74 (16.25)	6.28 (8.62)	5.47	(4.92 to 6.01)
After 99th percentile capping and excluding paid-for partners	11.40 (15.92)	6.28 (8.62)	5.11	(4.61 to 5.72)
After 99th percentile capping and adjusting for counting strategy			3.24	(2.81 to 3.68)
After 99th percentile capping and adjusting for sexual attitudes			4.31	(3.80 to 4.82)
After 99th percentile capping and adjusting for counting strategy and sexual attitudes			2.63	(2.22 to 3.04)
Unweighted, weighted denominator	6,028, 7,170	8,530, 7,323		

*p value for difference in means is < 0.001 for each row.

Over the past year, men reported an average of 1.27 partners and women 1.04, with a difference in means of 0.23 (95% confidence interval [CI]: 0.14 to 0.32). Over a five-year period, the mean number of partners reported by men was 2.66, compared with 1.84 for women, and the difference increased to 0.82 (95% CI: 0.55 to 1.09). Over the lifetime period, this difference increased substantially to 7.02 (4.97 to 9.08), with men reporting a mean of 14.14 lifetime partners and women 7.12.

To examine the impact of the gendered difference in understanding of what “counts” as a sexual partner, we added to the one-year count any oral-sex-only partners that had not been included in the count of sexual partners in the past year. This led to a small increase in the difference in mean number of partners in the past year between men and women, from 0.23 to 0.28. Excluding new partners where sex first occurred abroad (and where the partner was not a U.K. resident), and new partners from outside the United Kingdom but while in United Kingdom, caused a modest reduction in the difference for the past five years—from 0.82 to 0.67.

Over the lifetime, the gender gap of 7.02 reduced to 5.47 after capping partner number at the 99th percentile for each sex-specific distribution. Excluding the number of paid-for partners resulted in a further small reduction to 5.11.

Adjusting for counting strategy (having already capped at 99th percentile) reduced the gender gap in mean partner number from 5.47 to 3.24. Additionally adjusting for sexual attitudes further narrowed the gap to 2.63. In total, these three factors accounted for a mean difference of 4.39 lifetime partners (63% of the gender disparity).

### Differences in Reported Lifetime Partners by Age

**Figure 2.**
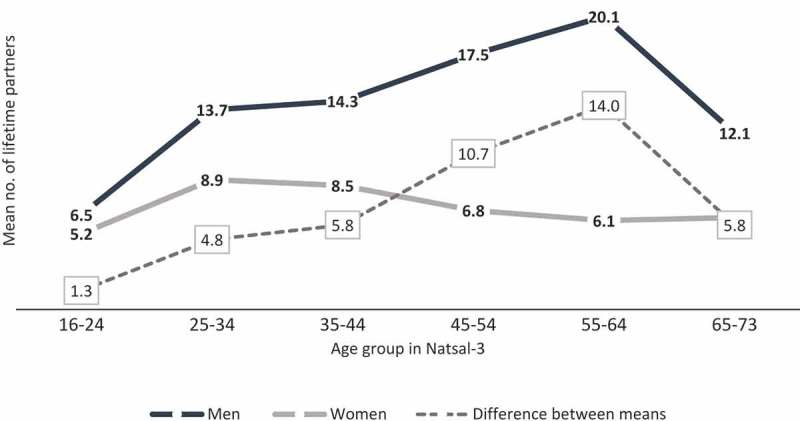
Mean number of lifetime partners (and difference between means) reported by men and women by age group in Natsal-3. Data originally published in Mercer et al. (2013).

Figure 2 shows the mean number of reported lifetime partners (and differences between means) by age group. In the youngest age group (16–24), the mean difference is 1.3, increasing steadily to 14 in the 55 to 64 age group and decreasing again to 5.8 in the oldest age group (65 to 73).

## Discussion

Our analysis of nationally representative data suggests that almost two-thirds of the gender gap in number of lifetime partners reported by men and women in Britain may be explained by three factors: a greater propensity among men to report extreme values; a greater propensity among women to count rather than estimate their lifetime partners; and gendered differences in attitudes toward casual and nonexclusive sex. The disparity seems barely affected by gender differences in the reported number of lifetime paid-for partners, but gender differences in reported non-U.K.-resident sexual partners have a modest (nonsignificant) impact in a five-year period and cannot be discounted as a potential explanation over the lifetime. Men were more likely than women to exclude oral-sex-only partners from their total count, and so adding them back in led to a slight (nonsignificant) widening of the gap.

The gender discrepancy is biggest over the lifetime period, both relative to the means and in absolute terms; in other words, errors appear to amplify over long time periods rather than gradually accumulate in proportion to time. Longer recall allows time for reevaluation of sexual histories in light of attitudes and experiences; and the larger the number, the harder it is to count. Given this, the increase in the gender gap by age group (Figure 2) is not surprising, because older participants have a larger number of years in which to accrue partners and have to recall the number over a longer period of time (age effects). Changes over time (period effects) may also provide an explanation, if over time it has become less “socially undesirable” for men to report few partners and for women to report many partners. The fact that the gender gap has narrowed in recent decades supports this hypothesis. In Figure 2, it is striking that the male mean increases by age group but the female mean does not. Figure 2 also shows that the gender discrepancy is largely driven by individuals in older age groups. However, it cannot shed light on whether differences are real or artifactual.

A key strength of this analysis is the large representative sample. Most studies of reporting bias in sexual behavior are limited to college students (Alexander & Fisher, 2003; Fisher, 2013; Jonason & Fisher, 2009) or high-risk populations (Brewer et al., 2000), and inherent sampling bias may mask or accentuate gender differences. Others manipulate reporting in laboratory conditions, which may not reflect how people respond in a real-life survey (e.g., Fisher, 2013). We were also able to consider a wide range of candidate explanations within the same data set, albeit not all over the lifetime period. A key limitation is that our analysis relied on assumptions, not all of which are testable. For instance, we capped extreme high values on the assumption that these overreported partners, but we were not able to test whether low values were underreports. Previous studies suggest that women increase their reported number of partners more than men when they think they are attached to a lie detector (Fisher, 2013), and studies of survey mode show that women increase their estimates of lifetime partners with Web versus telephone surveys, whereas men’s estimates stay constant (Brown et al., 2017). Similarly, we assumed that counting strategy and sexual attitudes influence the reported number of partners, when reverse causality (and bidirectionality) is also plausible. However, the noted (and other) studies that show how reporting is affected by manipulating survey conditions (Brown et al., 2017; Fisher, 2009, 2013) lend some support for this causal direction, instead of, or as well as, the other way around. Qualitative studies lend further support; in development work for the Natsal-2 survey, several participants described how they omitted certain partners from their total count due to shame or embarrassment (Mitchell et al., 2007).

Our finding that 99th percentile capping reduces the gender gap is consistent with previous research suggesting that the discrepancy is due in large part to the upper tail of the distribution (Morris, 1993). We also confirm other studies in finding that women were more likely to count and men to estimate, and that these differences in accounting strategies also explain some of the gender difference in mean number of partners (Brown et al., 2017; Brown & Sinclair, 1999). Previous studies suggest that part of the explanation may lie in a male propensity to include oral-sex-only partners (Jeannin et al., 1998), yet we found the reverse in our case (a male propensity to exclude these partners).This may be due to differences in the context and phrasing of questions to participants or, alternatively, differences in gendered definitions of sex across populations and cultures. We were not able to investigate all potential sampling explanations. Nonparticipation in the survey may provide a partial explanation, if this is related to sexual behavior differentially by gender. Paid-for partners may also have been underreported, but the effect of their exclusion was so small that this is unlikely to be a major explanation, even allowing for underreporting. Brewer and colleagues (2000) suggested that underrepresentation of female sex workers fully accounts for the discrepancy, but their estimate of sex-worker partners was derived from a high-risk nonprobability sample, and they appeared to assume all partners reported by sex workers were new clients; thus, they may have overestimated. We did not investigate the effect of age mixing because this element has previously been shown to be very small (Wadsworth et al., 1996), and there are unlikely to be significant numbers of reported partners outside of the ages 16 to 74 survey age range. The exclusions and adjustments we made to the estimates of lifetime partners still left 37% of the mean difference unexplained. It seems likely that explanations we were unable to test in the lifetime period—particularly non-U.K.-resident partners—might account for some of this difference. Our attitude measures were also rather limited proxies for false accommodation to gender norms and may have underestimated the magnitude of this explanation.

The fact that the gender gap in reported lifetime partners has narrowed over time potentially reflects greater accuracy in the reporting of sexual behavior in general, due to improved survey methodology and changes in social attitudes that are more gender equal and tolerant of diversity (Copas et al., 2002). Indeed, statistical comparisons across surveys suggest that measurement error in reporting lifetime partners may be no more than for other less sensitive survey measures (Hamilton & Morris, 2010). It is also worth noting that gender differences are small for recent, shorter time periods, and these are the periods commonly used in understanding STI risk and modeling transmission. Our data suggest there may be a residual effect of adherence to conservative sexual attitudes and that a male tendency to report extreme values and to estimate rather than count could be key explanations for the gender disparity in reported lifetime partners.

Privacy, confidentiality, and nonjudgmental wording are key to minimizing bias (Catania, Gibson, Marin, Coates, & Greenblatt, 1990; Kreuter, Presser, & Tourangeau, 2008). Survey instructions to encourage counting rather than estimating values, as well as a verification prompt for extreme value reports, could possibly improve results, but these changes would need to be weighed against the need to ensure ease of completion for participants and to avoid implying disbelief or judgment of answer.
